# Factors influencing loyalty to online health consultation platform: a cross-strait cultural perspective

**DOI:** 10.1186/s12913-023-09518-0

**Published:** 2023-05-23

**Authors:** Li-Yun Huang, Wen-Ming Shiau, Pei Chin Chou

**Affiliations:** 1 Division of Dermatology, E-Da Cancer Hospital, Kaohsiung, Taiwan; 2 Institute of Information Management, National Chung Cheng University, Chia-Yi, Taiwan; 3 Department of Respiratory Therapy, Fu Jen Catholic University, No.510, Zhongzheng Rd., Xinzhuang Dist, New Taipei City, 242062 Taiwan

**Keywords:** Online Health Consultation platform, Loyalty, Perceived Health Risks, Expectation confirmation theory, Trust, Culture

## Abstract

**Background:**

The geographical, cultural, and linguistic proximity between Taiwan and Mainland China has facilitated rapid growth of cross-strait interactions. Both countries have developed online health consultation platforms on the Internet for the public to access healthcare related information. This study examines factors that influence loyalty to a specific online health consultation platform (OHCP) from a cross-strait perspective.

**Methods:**

Based on the Expectation Confirmation Theory and the combined Trust, Perceived Health Risks and Culture, we examine factors that influence loyalty to OHCPs among cross-strait users by investigating the roles of trust, perceived health risks, and culture. Data was collected through a questionnaire survey.

**Results:**

The research models used provide a high-power explanation of loyalty to OHCPs. Results generally align with those of previous studies, with the exception of the relationships between Perceived Health Risks and Perceived Usefulness, Perceived Usefulness and Loyalty, Confirmation and Satisfaction, and Trust and Loyalty. In other words, culture may have moderated these relationships.

**Conclusions:**

Findings can help promote OHCPs among cross-strait users to make things easier for patients, and further reduce the load on the emergency department, especially in view of the still ongoing issues related to global outbreak of Coronavirus disease by facilitating early detection of potential cases.

**Supplementary Information:**

The online version contains supplementary material available at 10.1186/s12913-023-09518-0.

## Background

Online medical services help Internet users to manage healthcare for themselves or their families. Such online platforms are becoming the most trusted source of healthcare information, second only to physicians. A large number of Internet users now search for health-related information via the Internet [1–3]. Especially since 2019, the global outbreak of Coronavirus disease (COVID-19) has driven hospitals to adopt preventive measures and increase demand for online consultation services [4].

Many countries have developed online health consultation platforms (OHCPs) to meet the needs of their people. For example, the State Council of China vigorously developed the platform “Internet +” allowing novel online-offline interactions with greater efficiency and convenience. This Internet service was designed to improve efficiency in the use of medical resources, to reduce service costs, and to accelerate the development of new Internet services for medical care, healthcare and pensions [5]. Online medical services are developed at three levels: (a) online health consultation; (b) online remote consulting; and (c) online consulting to provide advice and medical information regarding treatments, prescriptions and disease prevention, under certain regulations. These services allow patients to seek medical diagnosis and guidance via computers or cellphones without having to travel to a clinic or hospital [6]. In Taiwan, Ministry of Health and Welfare has an OHCP that offers the public free consultation with physicians, pharmacists and nutritionists. The platform not only improves doctor-patient relationships, but also helps reduce unnecessary hospital visits, thereby saving medical and other resources [7]. During COVID-19 epidemic, Ministry of Health and Welfare expanded the scope of online diagnosis and treatment to meet the medical needs of people living in home isolation or quarantine [8].

The cross-strait interaction in economic, trade, social, cultural, and educational areas is frequent. According to 2019 Taiwan Medical Travel statistical reports, most of the international patients come from China (34.57%) [9]. Meanwhile, there were 404,000 Taiwanese working in Mainland China in 2018 [10] including businessmen and their families and they would use OHCPs of both sides. Therefore, comparing the loyalty in using OHCPs can provide information for both governments and platform providers as reference for improving the functioning and effectiveness of OHCPs.

### Model on the intention of continuous use of information system (IS)

Bhattacherjee [11] based on Oliver’s Expectation Confirmation Theory (ECT) model proposed the post-acceptance model of information system (IS) continuance which includes Perceived Usefulness, Confirmation, Satisfaction and Continuance Intention of IS.

In consumer terms, loyalty is the repurchasing behavior. The increasing popularity of the Internet and e-commerce has led to many studies on the so-called “e-loyalty” or e-commerce loyalty. Repeated visits to the same website is defined as website loyalty, reflecting customers’ willingness in maintain a stable future relationship with the site, and taking the site as their first choice when repurchasing a specific product or service online [12, 13]. We consider the intention to continue using the same online system a sign of loyalty. The major factors that affect loyalty are satisfaction [13–15], trust [14–16] and perceived usefulness [17]. Kim et al. [18] synthesized a model of consumer trust and satisfaction in the context of e-commerce, and indicate that trust has a longer-term impact on consumers’ e-loyalty through satisfaction. Dung [15] found that customer trust has a significant positive effect on customer satisfaction and loyalty, and customer satisfaction has significant positive effect on customer loyalty. Ayanso et al. added perceived risk to the model, and found that perceived usefulness and risk have a positive effect on satisfaction and further affect physicians’ intention to use electronic medical records [19]. Trust and perceived usefulness are also known to affect consumers’ acceptance and their continuing use of online medical services [20]. Sreelakshmi and Prathap [21] found that perceived susceptibility and perceived severity significantly affect perceived usefulness. This study is based on the post-acceptance model of IS continuance, and combines loyalty, trust, and perceived susceptibility and seriousness to explore factors influencing loyalty as displayed by continuous use of a given OHCP.

### National culture

While the language used in Taiwan and China is the same, there are cultural differences. Hofstede classified cultures at the national level in 6 aspects: power distance, uncertainty avoidance, individualism, masculinity, long term, and indulgence. Internet development differs across races and cultures. Erumban and De Jong [22] indicated that the national culture and the Information and Communications Technology (ICT) adoption rate of a country are closely related, and it appears that most of the Hofstede dimensions are important in influencing ICT adoption. Jauw and Purwanto [23] use cultural dimensions as a moderating variable to explore the effect of e-service quality on satisfaction with online purchases.

According to Hofstede [24], Taiwan and China have different cultures; Taiwan’s scores are lower on measures of power distance, individualism, and masculinity, but higher on measures of uncertainty avoidance and long-term orientation. Especially, the scores between Taiwan and China are largely different in the dimensions of power distance, masculinity, uncertainty avoidance, and indulgence. China’s culture has a high level of power distance [25]. Franque et al. [26] reported that the power distance had a significant moderating effect in relationship satisfaction and continuance intention. Chen et al. [27] and Lin and Ho [28] mainly focused on examining the influence of masculinity and uncertainty avoidance in cultures of Taiwan and China. Based on the related prior studies, we chose the four commonly used dimensions of Hofstede’s classification as culture dimensions for this study. Based on the aforementioned related studies we developed our research model.

## Methods

The study employed an anonymous online questionnaire, and the survey link was posted on various online health consultation platforms in both Taiwan and China. Ethical approval for the study was obtained from the Chang Gung Medical Foundation Institutional Review Board – IRB serial number: 201600336B0. The ethics committee approved this study, as it was deemed non-interventional and in compliance with national and international guidelines for research on humans. A statement was placed at the beginning of the questionnaire to obtain participants’ consent. The respondents who answered the questionnaire were de-identified to ensure anonymity and privacy.

### Research framework

**Fig. 1:**
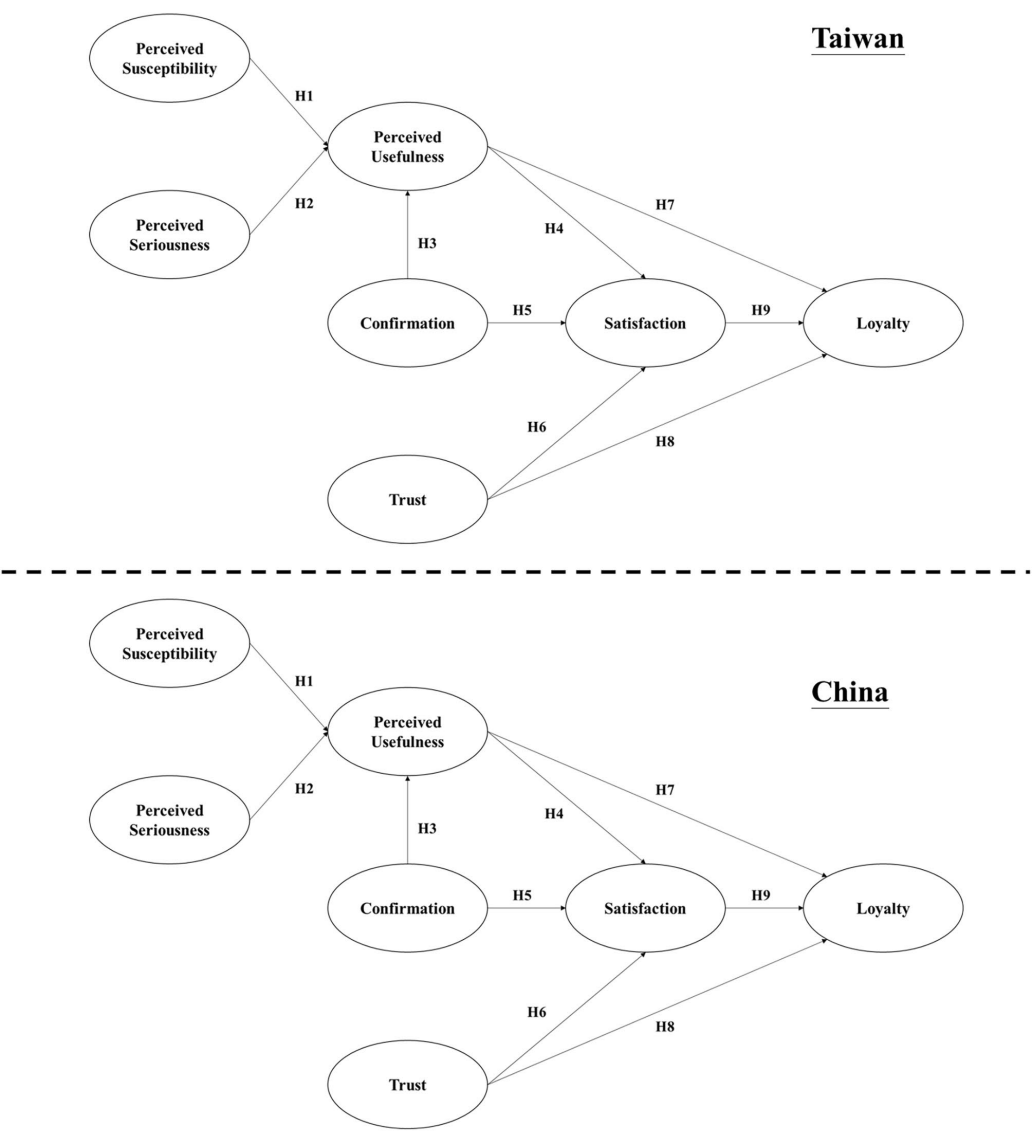
Research Framework

As aforementioned, research framework was developed by modifying the post-acceptance model of IS continuance through the addition of four variables: loyalty, trust, perceived susceptibility and seriousness, and a moderator, culture, to examine the differences between Taiwan and China. Since the moderator variable was expected to influence all the relationships between variables in the research framework, Fig. 1 illustrates the research framework into two groups. The data were grouped by culture (Taiwan or China) and research frameworks were tested accordingly. The following hypotheses were formulated based on the research framework.

H1: Perceived susceptibility has positive effects on people’s perceived usefulness of OHCP.

H2: Perceived severity has positive effects on people’s perceived usefulness of OHCP.

H3: Confirmation of people’s expectations of an OHCP has positive effects on people’s perceived usefulness of that system.

H4: People’s perceived usefulness of an OHCP has positive effects on their satisfaction with that system.

H5: Confirmation of people’s expectations of an OHCP has positive effects on their satisfaction with that system.

H6: People’s trust in an OHCP has positive effects on satisfaction with that system.

H7: People’s perceived usefulness of an OHCP has positive effects on their loyalty to that system.

H8: People’s trust on an OHCP has positive effects on their loyalty to that system.

H9: People’s satisfaction with an OHCP has positive effects on their loyalty to that system.

### Instrument development

The online questionnaire used for data collection was based on previous related studies with models having good reliability and validity scores. It consisted of 31 model-related items, and additional 5 items on demographic information like gender, age, educational level, commonly used health consultation platforms, and usual customary devices. Each item was measured on a 5-point Likert scale (from 5: strongly agree, to 1: strongly disagree). To assess the validity of the questionnaire, we invited 5 experts with expertise in healthcare information, culture, and online consulting platforms, including 3 industry experts (2 from China and 1 from Taiwan) with substantial experience in these areas, and 2 academic experts who have taught and published papers on culture and online consulting platforms. They were asked to review the research framework, the format of the measurement items, the wording of the scale items, and the length of the instrument to ensure the rigor of the research design, and the validity of the instrument. To avoid the non-response problem, several steps were taken to minimize the risk of sampling bias. Firstly, we targeted the users of OHCPs by publicizing the invitation on relevant websites from both Taiwan and China instead of general ones. Secondly, we adjusted the timing and frequency of sending out the survey questionnaire to increase the likelihood of participants responding. Lastly, we optimized the survey design with appropriate length and offered incentives to encourage participation.

Data were processed using the SPSS 18.0 for descriptive statistical analyses, two sample t-tests, and reliability/validity analyses. The SmartPLS 3.2.6 was used for partial least squares (PLS) including confirmatory factor analysis, confirmatory causal modeling, and hypothesis testing.

## Results

We received a total of 125 responses to our online questionnaires. After removing 10 responded questionnaires with excessive missing data, a total of 115 questionnaires were analyzed: 61 from Taiwan (53.3%) and 54 from China (47.0%). Taiwanese responders were mostly male (85.25%), aged 21 to 30 years old (65.57%), with college or university degrees (77.05%). The most popular OHCP in Taiwan was found to be the Kangjian Magazine (16.39%). The Chinese responders were mostly male (79.63%), aged 31 to 40 years old (50%), with college or university degrees (72.22%). The most popular OHCP in China was the HaoDF (29.63%) consultation platform. Table 1 shows the results of descriptive statistical analyses of the two samples.

**Table 1:** Basic sample information

Factor	Class	Taiwan	Percent	China	Percent
Gender	Male	52	85.25	43	79.63
	Female	9	14.75	11	20.37
Age	≤ 20	6	9.84	0	0
	21–30	40	65.57	16	29.63
	31–40	8	13.11	27	50
	41–50	2	3.28	10	18.52
	51–60	2	3.28	1	1.85
	≥ 60	3	4.92	0	0
Education	Junior high school	2	3.28	0	0
	High school	8	13.11	0	0
	Colleges & Universities	47	77.05	39	72.22
	Master or above	4	6.56	15	27.78
Commonly used Health consultation platform (Taiwan)	Kingnet Online Hospital	3	4.92		
	TAIWANeDOCTOR	2	3.28		
	Kangjian Magazine	10	16.39		
	5151 Online Health Care	5	8.19		
	Other	41	67.22		
Commonly used Health consultation platform (China)	HaoDF			16	29.63
	ChunYu			2	3.7
	DXY			18	33.33
	Baidu			11	20.37
	Good Doctor			4	7.41
	Other			3	5.56
Usual customary device	Computer	20	32.79	8	14.81
	Smart phone	35	57.38	46	85.19
	Both	6	9.83	0	0

Results of the two-sample t-test show the differences in Masculinity, Uncertainty Avoidance, and Individualism dimensions between the China and Taiwan samples. The level of power distance was not different between the two samples, however, China’s uncertainty avoidance score was higher than that of Taiwan which is different from Hofstede’s results. Meanwhile, the differences between Individualism in the two countries were trivial in Hofstede’s study. As previously mentioned, masculinity is a common dimension used in prior studies to verify the cultural differences between Taiwan and China. Based on this finding, we chose Masculinity as the culture factor for our study.

### Reliability and validity analysis

The result found few items with factor loadings of less than 0.5 or variance inflation factor (VIF) higher than 5. After removing these items, the related factor loadings were > 0.7 [29], Cronbach’s α was > 0.7 [30], composite reliability (CR) values were > 0.7 [29], average variance extracted (AVE) values of individual dimensions were all > 0.5 [29], and the square root of AVE was greater than relevant coefficients of other dimensions [29]. Following Hair et al. [29], factor loadings between 0.40 and 0.70 should be considered for removal, when deleting the indicator leads to an increase in the composite reliability. Since all CR values were > 0.7, indicators with factor loadings < 0.7 were not removed. In other words, questionnaire had reasonable reliability and internal consistency, and acceptable discriminability and convergent validity. Meanwhile, the VIF of each item is < 5 which explains that there is no collinearity between the variables.

### Analyses of the research model

SmartPLS 3.2.6 software was used for PLS analysis of paths between variables and determine interrelationships among factors and the explanatory power of the model. Bootstrap repeated sampling method (5,000 samples) [31] was used to estimate parameters and test the hypotheses.

**Fig. 2:**
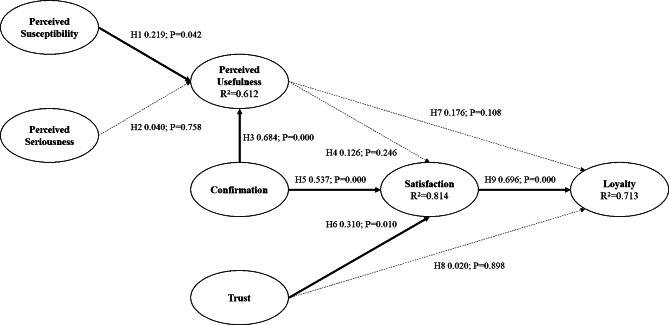
The results of Taiwan Group

For the Taiwan group, in the context of an OHCP, perceived susceptibility and confirmation were found to have positive effects on perceived usefulness with path coefficients and p-values of (0.684, p-value < 0.001) and (0.219, p-value < 0.050) respectively. Loyalty was found to have been influenced by satisfaction (0.696, p-value < 0.001). Trust and confirmation had positive effects on satisfaction with path coefficients and p-values of (0.310, p-value < 0.010) and (0.537, p-value < 0.001) respectively. However, perceived seriousness had no effect on perceived usefulness, and perceived usefulness had no effect on satisfaction. Furthermore, trust and perceived usefulness had no effect on loyalty. The model was able to explain 71.3% of the total variance of loyalty. The results are presented in Fig. 2; Table 2.

**Fig. 3:**
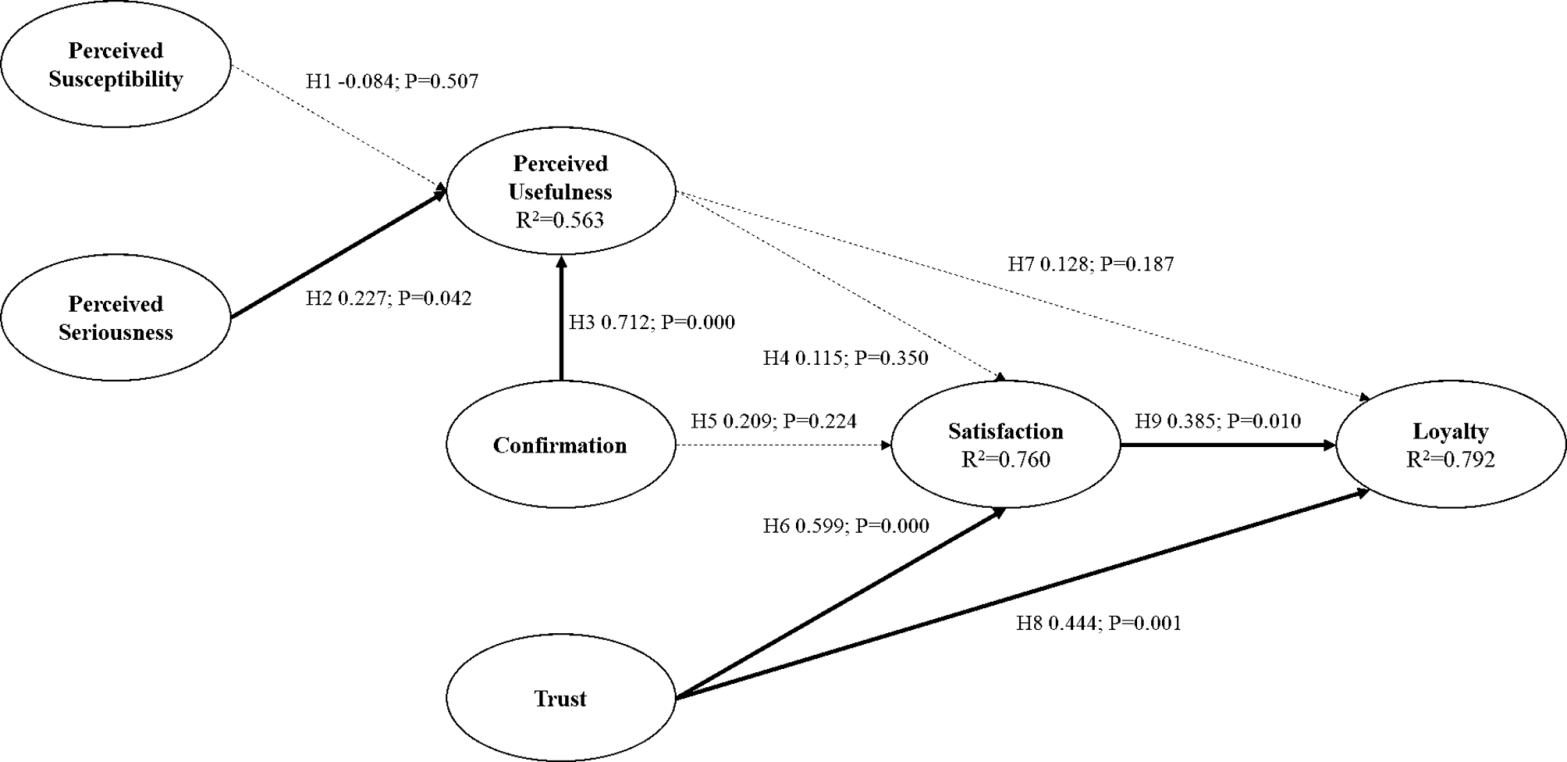
The Results of the China Group

For the China group, in the context of OHCP, perceived seriousness and confirmation had positive effects on perceived usefulness with coefficients and p-values of (0.227, p-value < 0.050) and (0.712, p-value < 0.001) respectively. Trust has a positive effect on satisfaction (0.599, p-value < 0.001). Loyalty was found to have been positively influenced by both satisfaction (0.385, p-value < 0.010) and trust (0.444, p-value < 0.001). However, perceived susceptibility had no effect on perceived usefulness. Furthermore, neither perceived usefulness nor confirmation had any effect on satisfaction, and perceived usefulness had no effect on loyalty. The model was able to explain 79.2% of the total variance of loyalty. Results are presented in Fig. 3; Table 2.

**Table 2:** Hypothesis testing results

Hypothesis		Taiwan Group			China Group		
		Path Coefficient	P value	Support	Path Coefficient	P value	Support
**H1**	PSu -> PU	0.219	0.042^*^	Y	-0.084	0.507	N
**H2**	PSe -> PU	0.040	0.758	N	0.227	0.042^*^	Y
**H3**	CON -> PU	0.684	0.000^***^	Y	0.712	0.000^***^	Y
**H4**	PU -> SAT	0.126	0.246	N	0.115	0.350	N
**H5**	CON -> SAT	0.537	0.000^***^	Y	0.209	0.224	N
**H6**	TRU -> SAT	0.310	0.010^**^	Y	0.599	0.000^***^	Y
**H7**	PU -> LOY	0.176	0.108	N	0.128	0.187	N
**H8**	TRU -> LOY	0.020	0.898	N	0.444	0.001^***^	Y
**H9**	SAT -> LOY	0.696	0.000^***^	Y	0.385	0.010^**^	Y

Note: (1)PSe: Perceived Seriousness; PSu: Perceived Susceptibility; PU: Perceived Usefulness; CON: Confirmation; TRU: Trust; SAT: Satisfaction; LOY: Loyalty; (2)^*^p<0.05; ^**^p<0.01; ^***^p<0.001

## Discussion and conclusion

In the context of the platform they used, both the Taiwanese and Chinese groups showed significant main effects of confirmation on perceived usefulness, trust on satisfaction, and satisfaction on loyalty. This result is in conformity with previous studies of technology acceptance or attrition theories such as Marinkovic and Kalinic’s findings that the trust in platform affects users’ satisfaction with that service [13] and Park et al.’s findings that consumer satisfaction with service has a positive effect on loyalty [14]. However, we found no effects of users’ perceived usefulness of OHCP on their satisfaction with the platform. This finding was not in line with Marinkovic and Kalinic who reported effects of perceived usefulness on satisfaction [13]. One reason for this discrepancy in results might be both groups gave similar ratings to the usefulness of the platform.

### Culture influences online health consultation platform of usage

The higher scores of Masculinity among Chinese group of OHCPs than those among Taiwanese group may explain the moderating effects of culture on the relationships between Perceived Susceptibility and Perceived Usefulness, Perceived Seriousness and Perceived Usefulness, Confirmation and Satisfaction, and Trust and Loyalty on the two sample groups. The results confirmed the finding of Teo and Huang [32] that cultural values orientation impacts individual perception in determining teachers’ technology acceptance. The results also confirmed the finding of Sarkar et al. [16] that culture is a significant moderator in the hypothesized relationships. Especially, culture has significant moderating effects on trust, user satisfaction, and behavior intention.

Hofstede’s research suggests that people in China tend to act in accordance with interests of the group rather that personal interest and may prioritize work over family and leisure. As a result, their perceptions of susceptibility to developing a health problem do not significantly affect their perceptions of usefulness of the OHCP. However, once they experience a health problem, they tend to perceive the OHCP as more useful if they perceive the problem to be more severe. Additionally, when they have a higher level of trust in the OHCP they are more likely to develop a higher level of loyalty towards the OHCP.

On the other hand, Taiwan people share long-term commitment with their close groups such as family members in a slightly feminine society and believe that the quality of life is a sign of success. As a result, Taiwan users are more likely to perceive the usefulness of the OHCP, when they perceive higher susceptibility to developing a health problem. After experiencing a health problem, regardless of whether perceived severity is high or low, they tend to find the OHCP useful. Meanwhile, the higher the level of confirmation they perceive about the OHCP, the higher is the satisfaction with the OHCP. In other words, the feminine tendency of Taiwanese people leads them to prioritize preventive healthcare and maintain the quality of life.

### Research limitations

This study collected data via online questionnaires. Since the questionnaire was self-reported by respondents, it is hard to confirm the authenticity of the respondents’ answers. As aforementioned, various measures were taken to reduce the risk of sampling bias. However, when conducting an online survey with a large number of anonymous participants, non-response can still be an issue. Specifically, in this study, fewer female respondents may indicate bias in sample recruitment and selection. Therefore, it is important to exercise caution when applying the results of this study.

### Research contributions

Our results provide OHCPs serving cross-strait clients a better understanding of ways that can help them retain users of their platforms. For managers of Taiwanese platforms, we recommend enhancing and increasing the functionality and quality of the platform to meet their users’ expectations and increase trust with platform.

For managers of platforms that serve Chinese users, we recommend focusing on users’ trust in the platform to increase user satisfaction with the system and further enhance loyalty to the platform. The trust variable in the OHCPs is defined as the user’s confidence in reliability and dependability of the OHCP and is measured through three items adopted from Valvi and West [4]. Our results suggest that OHCP providers should prioritize functional design integrity and platform operational stability to enhance user satisfaction. In particular, an increase in trust leads to increased loyalty among Chinese users towards the platform. In China, OHCPs are allowed to perform online triage and online consultations. Taiwan has stricter regulations on providing diagnoses online. Thus, providing similar online services for remote patients who have difficulty in accessing medical resources can definitely increase the usefulness of the OHCPS. This phenomenon has been accelerated by outbreak of the COVID-19 pandemic. OHCP providers are strengthening the consultation function through 5th generation wireless systems and provide stable face-to-face consultation services and further alleviate the workload of the emergency departments of hospitals.

Although the explaining power of our research model was up to 70%, there are other important factors that can be added to increase the explanatory power. Future studies can explore other factors affecting loyalty and cultural relationships between users and physicians, and use and combine other cultural dimensions or theories (e.g. GLOBE framework) to further promote users’ continued using and loyalty to OHCP. In addition, because Taiwan has highly accessible medical resources and a more convenient National Health Insurance system than China, the number of people in Taiwan who use OHCP is relatively limited. However, since COVID-19 outbreak, the government has gradually loosened telemedicine related regulations and therefore we also suggest future research to expand the sample sizes, and further enhance the representativeness of the sample.

## Electronic supplementary material

Below is the link to the electronic supplementary material.

Supplementary Material 1: Table S1. Items of the Questionnaire; Table S2. Two-sample t-test of culture dimension; Table S3. Reliability and Validity Analyses.

## Data Availability

The datasets generated and analyzed during the current study are not publicly available due to data of this study contain information that compromise the privacy of research participants but are available from the corresponding author on reasonable request.

## List of abbreviations

AVE: Average Variance Extracted
CON: Confirmation
CR: Composite Reliability
ECT: Expectation Confirmation Theory
ICT: Information and Communications Technology
IS: Information System
LOY: Loyalty
OHCPs: Online Health Consultation Platforms
PLS: Partial Least Squares
PSe: Perceived Seriousness
PSu: Perceived Susceptibility
PU: Perceived Usefulness
SAT: Satisfaction
TRU: Trust
VIF: Variance Inflation Factor
