# Supplementary material for: Factors influencing loyalty to online health consultation platform: a cross-strait cultural perspective
Source: BMC Health Serv Res. 2023 May 23;23:522. doi: 10.1186/s12913-023-09518-0 (PMC10204682; doi:10.1186/s12913-023-09518-0)
Supplement: Supplementary file 1 — Supplementary Material 1: Table S1. Items of the Questionnaire; Table S2. Two-sample t-test of culture dimension; Table S3. Reliability and Validity Analyses. [file 12913_2023_9518_MOESM1_ESM.docx]

**Supplementary Materials**

**Table S1. Items of the Questionnaire.**

| **Perceived Susceptibility [1]** |
| --- |
| It is very unlikely I will get disease in the future. |
| I feel I might get disease in the future. |
| There is a good possibility I will get disease in the next 10 years. |
| My chances of getting disease are high. |
| I have very high possibility than the average people to get disease. |
| **Perceived Severity [1]** |
| The thought of disease scares me. |
| When I think about disease, my heart beats faster. |
| I am afraid to think about disease. |
| The fact that I would experience with disease would last a long time. |
| Disease would threaten a relationship with my partner. |
| If I had disease, my whole life would change. |
| If I develop disease, I would not live longer than 5 years. |
| **Perceived usefulness [2]** |
| The online health consultation platform can be useful in managing my daily health. |
| The online health consultation platform can be beneﬁcial to me. |
| The online health consultation platform can be valuable to my healthcare. |
| The online health consultation platform can be advantageous in better managing my health. |
| **Confirmation [3]** |
| My experience with using online health consultation platform was better than expected. |
| The service level provided by online health consultation platform was better than expected. |
| Overall, most of my expectations from using online health consultation platform were confirmed. |
| **Trust [4]** |
| This online health consultation platform can successfully solve my problem. |
| I can trust the performance of this online health consultation platform to be good |
| This online health consultation platform is reliable. |
| **Satisfaction [5]** |
| This online health consultation platform meets my needs. |
| This online health consultation platform is very competent. |
| This online health consultation platform gives me the service that I expect. |
| This online health consultation platform gives an excellent service. |
| My experience with the online health consultation platform is positive. |
| **Loyalty [5]** |
| I would recommend online health consultation platform to my relatives and friends. |
| I will choose online health consultation platform in the future to consult my disease. |
| The online health consultation platform is my first choice. |
| I will say positive things about online health consultation platform. |

Reference

1. Noroozi A, Jomand T, Tahmasebi R. Determinants of breast self-examination performance among Iranian women: an application of the health belief model. Journal of Cancer Education 2011; 26(2): 365-374.
2. Lim S, Xue L, Yen CC, et al. A study on Singaporean women's acceptance of using mobile phones to seek health infor-mation. International journal of medical informatics 2011; 80(12): e189-e202.
3. Bhattacherjee A. Understanding information systems continuance: An expectation-confirmation model. MIS quarterly 2001; 25(3): 351-370.
4. Valvi AC, West DC. E-loyalty is not all about trust, price also matters: extending expectation-confirmation theory in bookselling websites. Journal of Electronic Commerce Research 2013; 14(1): 99.
5. Lin, CH, Wu CW, Cheng YH. The empirical study of consumers' loyalty for display technology. Journal of Business Re-search 2015; 68(11): 2260-2265.

**Table S2. Two-sample t-test of culture dimension.**

| **Culture Dimension** | **t-test (df)** | **p-value** |
| --- | --- | --- |
| **Power Distance** | 1.534(113) | 0.128 |
| **Uncertainty Avoidance** | -2.815 (113) | 0.006* |
| **Masculinity** | -3.716 (113) | 0.000* |
| **Individualism** | -2.852 (113) | 0.005* |

**Note: *p< 0.05**

**Table S3. Reliability and Validity Analyses**

|  | **Item** | **Taiwan Group** | | | | | **China Group** | | | | |
| --- | --- | --- | --- | --- | --- | --- | --- | --- | --- | --- | --- |
|  |  | **FA** | **VIF** | **AVE** | **CR** | **CA** | **FA** | **VIF** | **AVE** | **CR** | **CA** |
| **PSe** | PSe_1 | 0.795 | 2.264 | 0.609 | 0.903 | 0.896 | 0.744 | 1.769 | 0.596 | 0.898 | 0.865 |
|  | PSe_2 | 0.787 | 4.372 |  |  |  | 0.690 | 1.574 |  |  |  |
|  | PSe_3 | 0.677 | 3.686 |  |  |  | 0.761 | 2.043 |  |  |  |
|  | PSe_4 | 0.792 | 1.861 |  |  |  | 0.763 | 2.029 |  |  |  |
|  | PSe_5 | 0.761 | 2.380 |  |  |  | 0.812 | 2.412 |  |  |  |
|  | PSe_6 | 0.859 | 1.779 |  |  |  | 0.850 | 2.146 |  |  |  |
| **PSu** | PSu_1 | 0.957 | 2.223 | 0.584 | 0.842 | 0.833 | 0.903 | 2.958 | 0.735 | 0.917 | 0.884 |
|  | PSu_3 | 0.847 | 1.963 |  |  |  | 0.844 | 1.942 |  |  |  |
|  | PSu_4 | 0.673 | 3.049 |  |  |  | 0.845 | 3.001 |  |  |  |
|  | PSu_5 | 0.500 | 2.552 |  |  |  | 0.836 | 2.358 |  |  |  |
| **PU** | PU_1 | - | - | 0.892 | 0.961 | 0.940 | 0.898 | 2.818 | 0.853 | 0.946 | 0.914 |
|  | PU_2 | 0.935 | 3.696 |  |  |  | - | - |  |  |  |
|  | PU_3 | 0.952 | 4.842 |  |  |  | 0.946 | 4.195 |  |  |  |
|  | PU_4 | 0.947 | 4.577 |  |  |  | 0.926 | 3.226 |  |  |  |
| **CON** | Conf_1 | 0.895 | 2.774 | 0.809 | 0.927 | 0.882 | 0.932 | 3.410 | 0.881 | 0.957 | 0.933 |
|  | Conf_2 | 0.931 | 3.446 |  |  |  | 0.937 | 4.204 |  |  |  |
|  | Conf_3 | 0.872 | 2.095 |  |  |  | 0.947 | 4.297 |  |  |  |
| **SAT** | SAT_1 | 0.870 | 2.530 | 0.789 | 0.937 | 0.911 | 0.857 | 2.408 | 0.804 | 0.942 | 0.918 |
|  | SAT_3 | 0.906 | 3.250 |  |  |  | 0.920 | 4.413 |  |  |  |
|  | SAT_4 | 0.887 | 2.932 |  |  |  | 0.933 | 4.958 |  |  |  |
|  | SAT_5 | 0.890 | 2.735 |  |  |  | 0.874 | 2.421 |  |  |  |
| **TRU** | Trust_1 | 0.812 | 1.596 | 0.772 | 0.910 | 0.850 | 0.927 | 2.984 | 0.838 | 0.940 | 0.904 |
|  | Trust_2 | 0.913 | 3.063 |  |  |  | 0.922 | 3.178 |  |  |  |
|  | Trust_3 | 0.907 | 2.949 |  |  |  | 0.897 | 2.629 |  |  |  |
| **LOY** | Loy_1 | 0.872 | 1.909 | 0.751 | 0.900 | 0.835 | 0.885 | 2.260 | 0.800 | 0.923 | 0.875 |
|  | Loy_3 | 0.814 | 1.868 |  |  |  | 0.875 | 2.264 |  |  |  |
|  | Loy_4 | 0.912 | 2.561 |  |  |  | 0.923 | 2.953 |  |  |  |

Note: PSe: perceived seriousness; PSu: Perceived Susceptibility; PU: Perceived Usefulness; CON: Confirmation; TRU: Trust; SAT: Satisfaction; LOY: Loyalty.
